# Macromolecular crowding and bicarbonate enhance the hydrogen peroxide-induced inactivation of glyceraldehyde-3-phosphate dehydrogenase

**DOI:** 10.1042/BCJ20240597

**Published:** 2024-12-04

**Authors:** Rebecca H. J. Bloemen, Rafael Radi, Michael J. Davies, Eduardo Fuentes-Lemus

**Affiliations:** 1 Department of Biomedical Sciences, Panum Institute, University of Copenhagen, Blegdamsvej 3, Copenhagen 2200, Denmark; 2 Departamento de Bioquímica, Facultad de Medicina, Universidad de la República, Montevideo 11800, Uruguay; 3 Centro de Investigaciones Biomédicas (CEINBIO), Facultad de Medicina, Universidad de la República, Montevideo 11800, Uruguay; 4 Departamento de Química Física, Facultad de Química y de Farmacia, Pontificia Universidad Católica de Chile, Santiago, Chile

**Keywords:** glyceraldehyde-3-phosphate dehydrogenase, hydrogen peroxide, macromolecular crowding, peroxymonocarbonate, protein oxidation, redox regulation

## Abstract

The active site Cys residue in glyceraldehyde-3-phosphate dehydrogenase (GAPDH) is sensitive to oxidation by hydrogen peroxide (H_2_O_2_), with this resulting in enzyme inactivation. This re-routes the carbon flux from glycolysis to the pentose phosphate pathway favoring the formation of NADPH and synthetic intermediates required for antioxidant defense and repair systems. Consequently, GAPDH inactivation serves as a redox switch for metabolic adaptation under conditions of oxidative stress. However, there is a major knowledge gap as to how GAPDH is efficiently oxidized and inactivated, when the increase in intracellular H_2_O_2_ is modest, and there is a high concentration of alternative (non-signaling) thiols and efficient peroxide removing systems. We have therefore explored whether GAPDH inactivation is enhanced by two factors of *in vivo* relevance: macromolecular crowding, an inherent property of biological environments, and the presence of bicarbonate, an abundant biological buffer. Bicarbonate is already known to modulate H_2_O_2_ metabolism via formation of peroxymonocarbonate. GAPDH activity was assessed in experiments with low doses of H_2_O_2_ under both dilute and crowded conditions (induced by inert high molecular mass polymers and small molecules), in both the absence and presence of 25 mM sodium bicarbonate. H_2_O_2_-induced inactivation of GAPDH was observed to be significantly enhanced under macromolecular crowding conditions, with bicarbonate having an additional effect. These data strongly suggest that these two factors are of major importance in redox switch mechanisms involving GAPDH (and possibly other thiol-dependent systems) within the cellular environment.

## Introduction

Glyceraldehyde-3-phosphate dehydrogenase (GAPDH) is an asymmetric homotetrameric NAD^+^-dependent enzyme that catalyzes the oxidative phosphorylation of glyceraldehyde-3-phosphate (G3P) to 1,3-biphosphoglycerate, with concurrent formation of NADH. Together with the glycolytic role of GAPDH, this abundant cytosolic enzyme (c.f. estimated intracellular levels are low micromolar but can reach up to 75 µM in skeletal muscle cells [1]) has other non-glycolytic (moonlighting) activities in different cell organelles, affecting, amongst others, mRNA stability, cell trafficking and gene regulation [2]. Recent attention has focused on the role of GAPDH as a reversible metabolic switch in cells exposed to elevated (supraphysiological) steady-state levels of oxidants (e.g. cancer cells, or cells exposed to oxidative insults) [3,4]. GAPDH is particularly sensitive to oxidative post-translational modification by a number of oxidants including the two-electron oxidant, and signaling molecule hydrogen peroxide (H_2_O_2_) [5]. This inactivation arises from modification of the active site cysteine (Cys, position 152 in the human sequence, position 150 in the rabbit isoform), though alteration of other residues, such as a nearby Cys (Cys_154_ in the human isoform), can also modulate its enzymatic activity [6,7]. Inactivation of GAPDH modulates its function (e.g. switching from a dehydrogenase to an acyl phosphatase activity [8]) and triggers the re-routing of the carbon flux from glycolysis to the oxidative phase of the pentose phosphate pathway (oxPPP). The oxPPP pathway is essential for the reduction of NADP^+^ to NADPH which is an essential reducing cofactor for the glutathione reductase, glutaredoxin and thioredoxin enzyme systems that play a key role in cellular defenses against oxidative stress [3]. Therefore, rapid and efficient H_2_O_2_-induced modification of GAPDH is essential for cellular signaling of cellular stress, and metabolic adaptation to these conditions.

Despite the strong evidence supporting an important role of GAPDH oxidation as a metabolic switch, a fundamental question remains as to how H_2_O_2_ achieves this against a high background of other thiols (typically high mM) and multiple efficient enzymatic peroxide-removing systems. Thus, whilst the second-order rate constant for the reaction of peroxiredoxins with H_2_O_2_ is 10^5^–10^8^ M^−1^ s^−1^ (depending on the isoform [9]), the reported rate constant for GAPDH with H_2_O_2_ is ∼7 M^−1 ^s^−1^ at pH 7.4 and 20°C [10], and 9.4 M^−1^ s^−1^ at pH 7.0 [11]. Although previous studies have reported a higher (but possibly erroneous) second-order rate constant in the order of 10^2^–10^3^ M^−1^ s^−1^ for the reaction between H_2_O_2_ and GAPDH [7,12], the former values are only slightly higher than that for the abundant (2–10 mM) antioxidant peptide glutathione (GSH) (rate constant, *k*, for reaction with H_2_O_2_ ∼0.9 M^−1^ s^−1^ at pH 7.4 and 37°C [12]). Thus, on pure kinetic grounds, it would not be expected that GAPDH would compete effectively for H_2_O_2_. To (partly) explain this conundrum, it has been reported that bicarbonate, an important biological buffer in mammalian cells and in equilibrium with CO_2_ (eqn 1), is required for the rapid inactivation of GAPDH. This is because both H_2_O_2_ and its conjugated base, ${\mathrm{HO}}_{2}^{-}$, react with CO_2_ to generate the powerful oxidant peroxymonocarbonate (${\mathrm{HCO}}_{4}^{-}$) (eqns 2 and 3), which reacts with GAPDH with a reported rate constant of ∼47 M^−1^ s^−1^ at pH 7.4 and 20°C [10]. Thus, inclusion of 25 mM bicarbonate in the reaction buffer enhances the rate of H_2_O_2_-mediated GAPDH inactivation, when compared with its absence. However, even with this (modest) increase in rate constant, the reactivity of GAPDH towards H_2_O_2_ is still orders of magnitude slower than for many peroxidase enzymes, suggesting that other factors must also play a role.

1 $$H^{+}+{\mathrm{HCO}}_{3}^{-}⇌CO_{2}(\mathrm{aq})+H_{2}O$$

2 $$H_{2}O_{2}+CO_{2}(\mathrm{aq})⇌H_{2}CO_{4}⇌H^{+}+{\mathrm{HCO}}_{4}^{-}$$

3 $${\mathrm{HO}}_{2}^{-}+CO_{2}(\mathrm{aq})⇌H_{2}{\mathrm{CO}}_{4}^{-}$$

We have recently proposed, and provided experimental data to support, the notion that macromolecular crowding is an important modulator of the reactivity of proteins with oxidants (including H_2_O_2_) and the mechanisms of these reactions [13–15]. This is logical, as crowding is an inherent property of most biological systems, and is known to modulate protein structure (e.g. folding and surface dynamics), protein-protein interactions, protein-substrate and protein-cofactor interactions, as well as the frequency of encounters between (macro)molecules and enzymes with substrates [14,16]. In this context, we have recently reported that macromolecular crowding results in an altered pattern of oxidation products formed on GAPDH treated with H_2_O_2_, with higher yields of irreversible products detected under crowded conditions when compared with dilute solutions [17]. Nevertheless, to the best of our knowledge, there is little data as to whether macromolecular crowding affects the extent and rate of H_2_O_2_-induced GAPDH inactivation (i.e. activity data).

In the light of these data, we hypothesized that a combination of both macromolecular crowding and the presence of bicarbonate, might have additive or synergistic effects on H_2_O_2_-induced inactivation of GAPDH. This hypothesis was tested in studies using rabbit muscle GAPDH (which has 95% sequence homology with the human isoform and a conserved catalytic site [18]), with this exposed to physiologically-relevant H_2_O_2_ doses in the absence or presence of different crowding agents (both small molecule and high molecular mass polymers with well-known physical and chemical properties [19]), and also in the absence or presence of 25 mM bicarbonate (and therefore physiological levels of CO_2_).

## Results

### Effect of macromolecular crowding on the H_2_O_2_-induced inactivation of GAPDH

**Figure 1.**
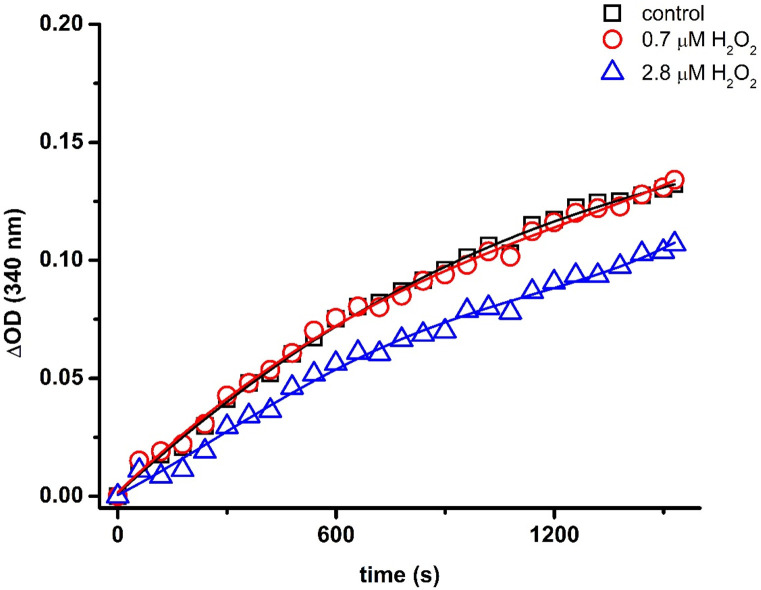
Changes in absorbance (ΔOD) at 340 nm, arising from NADH formation during the incubation of 0.2 mM G3P and 0.5 mM NAD^+^ with GAPDH samples pre-incubated in the absence or the presence of increasing doses of H_2_O_2_. GAPDH (0.8 μM, monomer) was incubated with 0, 0.7 or 2.8 μM H2O2 for 3 h at 37°C in 100 mM sodium phosphate buffer (pH 7.4) containing 0.1 mM DTPA before dilution (1:1) in 100 mM Tris buffer (pH 8.0) containing 0.4 mM G3P, 1 mM NAD^+^, 15 mM sodium arsenate and 0.2 mM EDTA. Representative data from one of six independent experiments.

Initial studies examined the effects of macromolecular crowding alone on H_2_O_2_-induced inactivation of GAPDH, with these data compared with dilute solutions (i.e. 100 mM phosphate buffer solution containing 0.1 mM DTPA). The effect of H_2_O_2_ on GAPDH activity (0.8 µM monomer units) was determined after the pre-incubation of the enzyme in the absence or presence of 0.35, 0.7, 1.4 and 2.8 µM H_2_O_2_ for 3 h at 37°C (oxidant-to-protein molar excesses of ∼0.4 ∼0.9, ∼1.8 and ∼3.5 respectively; concentrations and molar ratios likely to reflect the physiological to pathological range). A steady increase in absorbance at 340 nm, due to reduction of NAD^+^ to NADH), was observed over time on incubation with 0.2 mM G3P and 0.5 mM NAD^+^ (Figure 1). A similar kinetic profile was observed for GAPDH pre-incubated with 0.7 µM H_2_O_2_ under the same conditions. In contrast, pre-incubation with 2.8 µM H_2_O_2_ resulted in a significant decrease in GAPDH activity. This effect was confirmed by determining the initial slopes of the kinetic profiles, from which the GAPDH activity was determined relative to the controls (*vide infra*).

**Figure 2.**
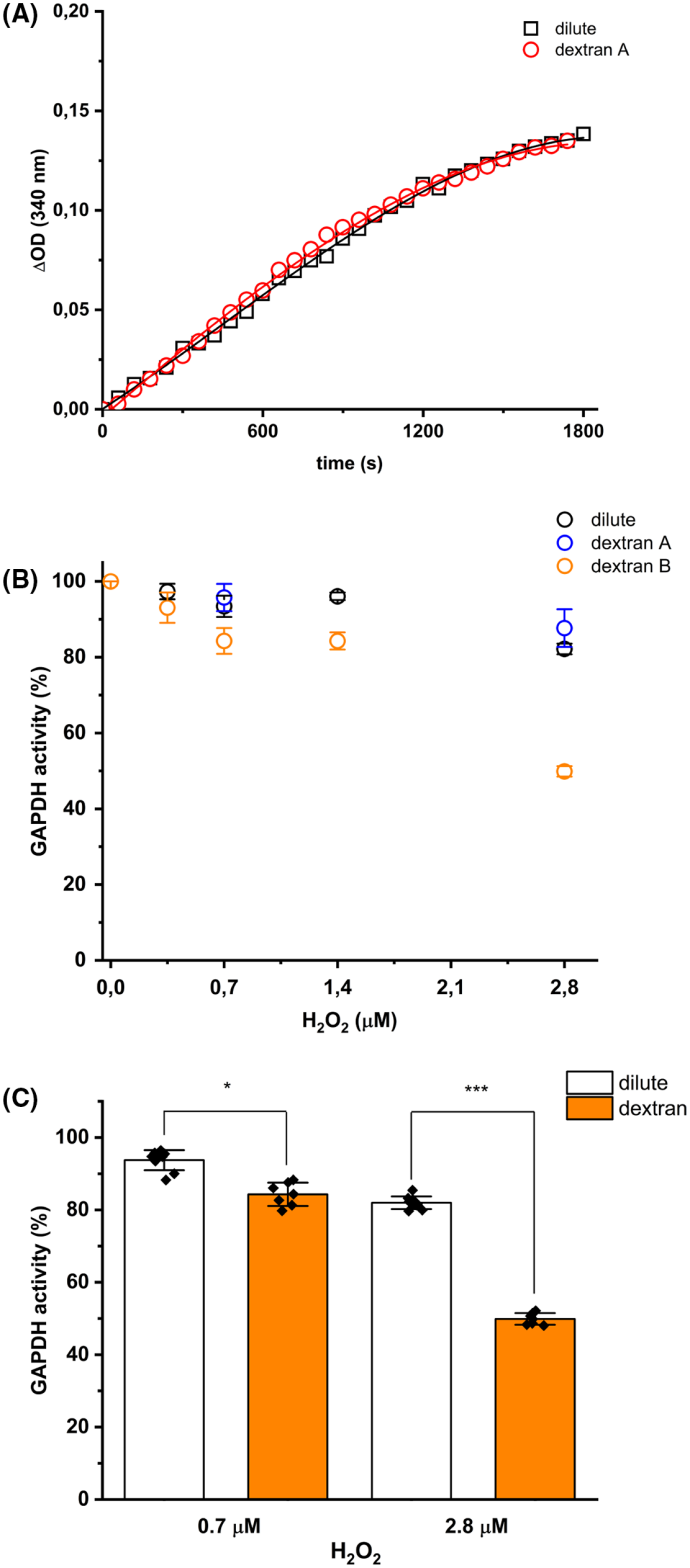
Effect of dextran on H_2_O_2_-mediated inactivation of GAPDH. (**A**) Changes in absorbance (ΔOD) at 340 nm, arising from NADH formation during the incubation of control (non-oxidized) GAPDH (0.4 μM) with 0.2 mM G3P and 0.5 mM NAD^+^ in the absence or the presence of 60 mg ml^−1^ dextran 35 present during the measurement of enzyme activity (dextran condition A). (**B**) GAPDH (0.8 μM, monomer) was incubated with 0, 0.35, 0.7, 1.4 or 2.8 μM H_2_O_2_ for 3 h at 37°C in either 100 mM sodium phosphate buffer (pH 7.4) containing 0.1 mM DTPA (dilute condition, black symbols), or in 120 mg ml^−1^ dextran in 100 mM sodium phosphate buffer (pH 7.4) containing 0.1 mM DTPA (dextran condition B; orange symbols) before measurement of enzyme activity (see Materials and methods). In addition, the activity of GAPDH samples pre-incubated under dilute conditions with varying H_2_O_2_ doses were subsequently measured in the presence of 60 mg ml^−1^ dextran 35 (crowded condition A; blue symbols). (**C**) Histogram and quantification of residual GAPDH activity expressed as a % of the incubated untreated control condition (0 μM H_2_O_2_) after incubation with 0.7 or 2.8 μM H_2_O_2_ for 3 h at 37°C under dilute (white bars) and dextran condition B (orange bars). Data are mean ± SD from at least six independent experiments, with statistical differences (as determined by one-way ANOVA and Dunnett's *post hoc* test) indicated as follows: *P < 0.05, **P < 0.01, and ***P < 0.001.

A previous study has reported that macromolecular crowding does not alter the specific activity of GAPDH, and only the monomer:dimer:tetramer equilibrium [20]. To confirm these activity data, we determined the effect of the crowding agent dextran 35 on enzyme activity. The presence of 60 mg ml^−1^ dextran 35 (at the time point at which activity was determined) did not alter the time course of NAD^+^ reduction, when compared with data obtained under dilute conditions (Figure 2A). This was confirmed for GAPDH samples pre-incubated with 0.7 and 2.8 µM H_2_O_2_ under dilute conditions, with the activity measurements recorded in the absence or the presence of 60 mg ml^−1^ dextran (Figure 2B). However, when the oxidation of GAPDH was carried out under crowded conditions (i.e. in the presence of 120 mg ml^−1^ dextran 35), an enhanced inactivation of the enzyme was determined (Figure 2B). Incubation of GAPDH with 0.7 µM H_2_O_2_ under dilute conditions resulted in a loss of ∼7% of the activity compared with the activity of the native, non-oxidized, enzyme, whilst incubation with 0.7 µM H_2_O_2_ in the presence of 120 mg ml^−1^ dextran 35 resulted in a ∼16% decrease in activity (Figure 2B,C). The effect of dextran 35 on H_2_O_2_-mediated inactivation of GAPDH was more pronounced with a 3.5-fold molar excess of H_2_O_2_ over protein concentration. A residual activity of ∼82 ± 1.4% (i.e. ∼18% loss of the activity) was determined under dilute conditions, whereas a residual activity of 50 ± 1.4% was determined for GAPDH samples incubated with 2.8 µM H_2_O_2_ under crowded conditions (Figure 2C).

**Figure 3.**
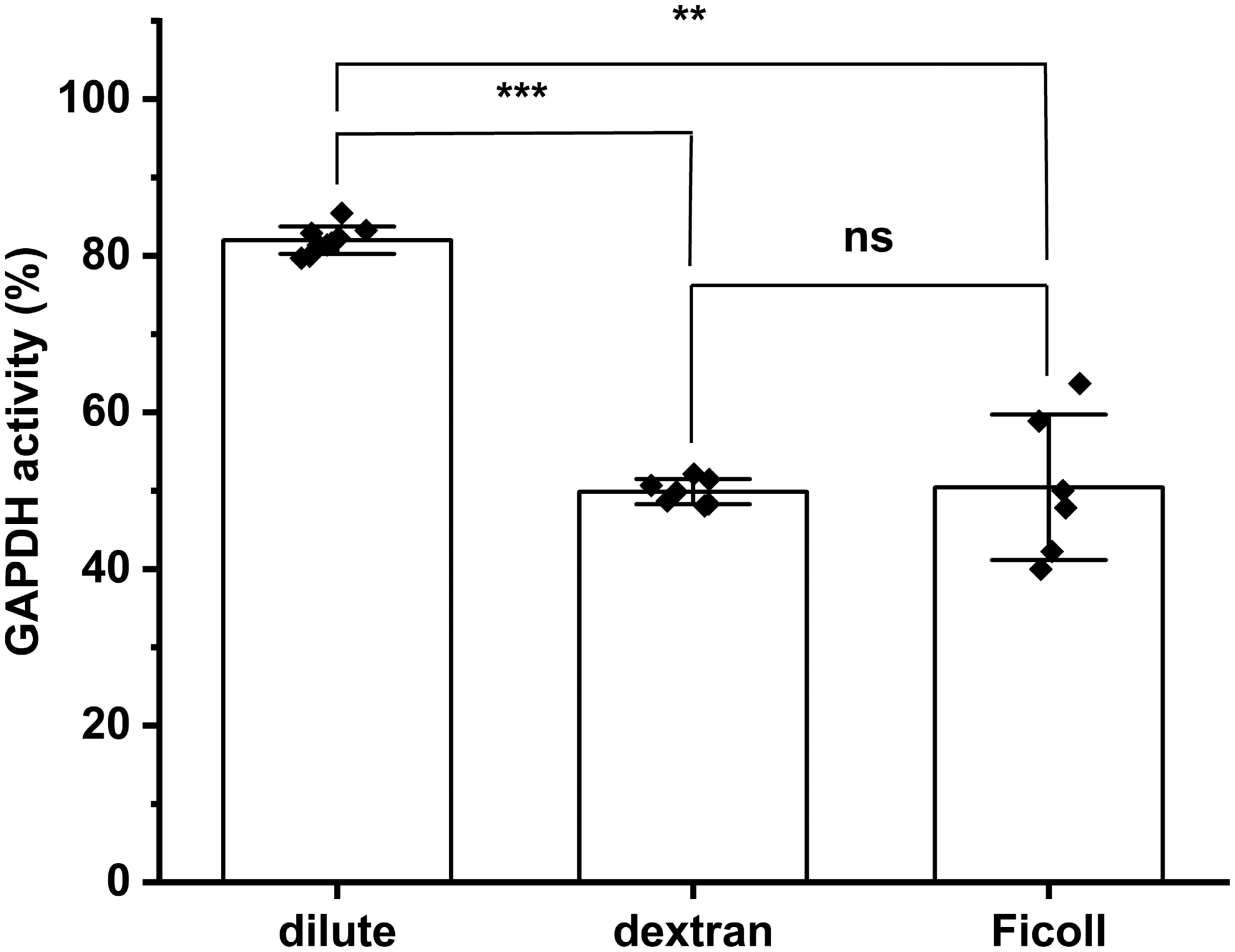
The enhancement of H_2_O_2_-mediated inactivation of GAPDH under macromolecular crowding conditions is independent of the macromolecular crowding agent utilized. GAPDH (0.8 μM, monomer) was incubated with 2.8 μM H_2_O_2_ for 3 h at 37°C under dilute (100 mM sodium phosphate buffer (pH 7.4) containing 0.1 mM DTPA), or crowded (buffer containing 120 mg ml^−1^ of either dextran 35 or Ficoll 70) conditions. GAPDH activity expressed as a % of the incubated untreated control condition (0 μM H_2_O_2_) was determined as described in the Materials and methods section. Data are mean ± SD from at least six independent experiments, with statistical differences (as determined by one-way ANOVA and Dunnett's *post hoc* test) indicated as follows: ns, no statistical difference, *P < 0.05, **P < 0.01, and ***P < 0.001.

To investigate whether this effect was specific to this linear polymer, or was a general effect of macromolecular crowding, similar experiments were carried out with the branched polymer Ficoll 70. The presence of Ficoll during the measurement of GAPDH activity did not alter the kinetic profiles (data not shown). However, oxidation of GAPDH with 2.8 µM H_2_O_2_ in the presence of 120 mg ml^−1^ Ficoll 70 resulted in a significantly greater loss of activity than under dilute conditions (Figure 3). A residual activity of 50 ± 9.1% was determined under these conditions, with this data showing no statistical differences when compared with the data obtained with dextran 35 under identical conditions, suggesting that this is a general crowding effect of these macromolecules, rather than a structure-dependent activity.

**Figure 4.**
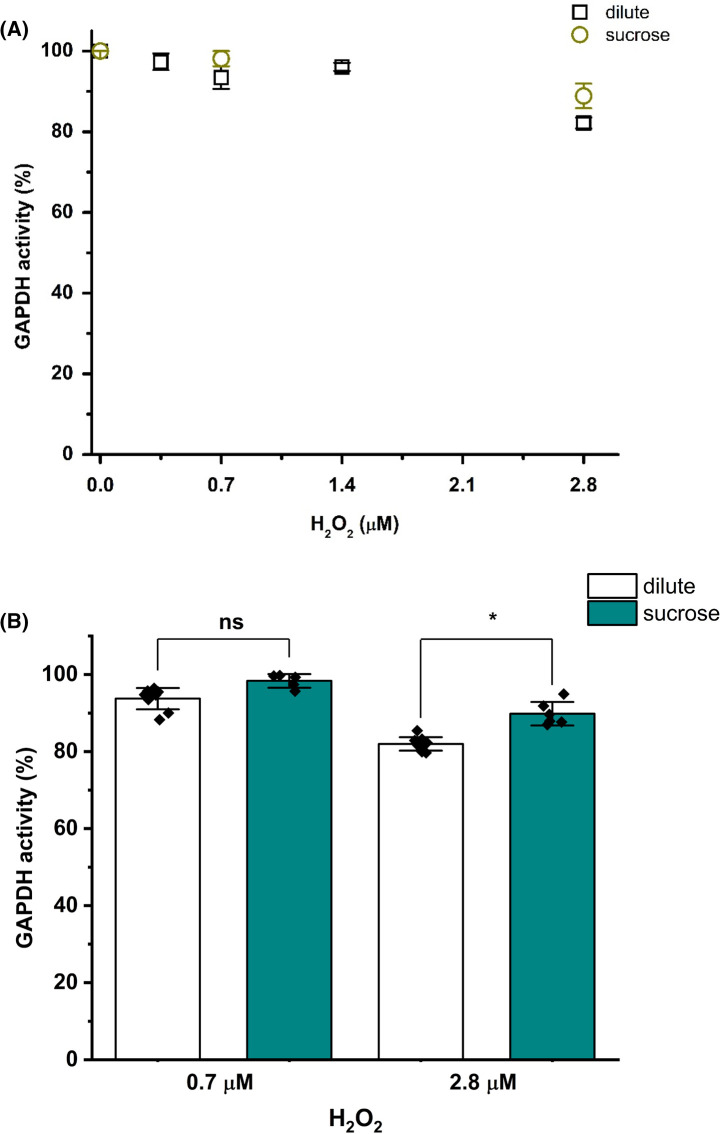
The low molecular mass crowding agent sucrose does not enhance H_2_O_2_-mediated inactivation of GAPDH. GAPDH (0.8 μM, monomer) was incubated with 0, 0.7 or 2.8 μM H_2_O_2_ for 3 h at 37°C in the absence or the presence of 120 mg ml^−1^ sucrose in 100 mM sodium phosphate buffer (pH 7.4) containing 0.1 mM DTPA. (**A**) GAPDH activity after incubation with H_2_O_2_ in the presence of sucrose. (**B**) Quantification of residual GAPDH activity after incubation with 0.7 or 2.8 μM H_2_O_2_ for 3 h at 37°C under dilute (white bars) and sucrose (dark yellow bars) conditions. Data are mean ± SD from at least six independent experiments, with statistical differences (as determined by one-way ANOVA and Dunnett's *post hoc* test) indicated as follows: ns, no statistical difference, *P < 0.05, **P < 0.01, and ***P < 0.001.

The effect of small molecules, which increase solution viscosity, but which do not have the space filling (exclusion phenomenon) effect of macromolecules such as dextran or Ficoll was also examined. Initial experiments with glycerol (120 mg ml^−1^) induced a complete loss of GAPDH activity when added to the measurement buffer (data not shown). This is ascribed to a protein unfolding activity of this compound. In contrast, the presence of 120 mg ml^−1^ sucrose during activity measurements did not induce any significant effects on enzyme activity relative to dilute conditions (data not shown). In contrast with the data obtained with the macromolecule crowding agents, incubation of GAPDH with increasing concentrations of H_2_O_2_ in the presence of 120 mg ml^−1^ sucrose did not enhance oxidant-induced inactivation of GAPDH (Figure 4).

### Effect of bicarbonate on H_2_O_2_-induced inactivation of GAPDH

**Figure 5.**
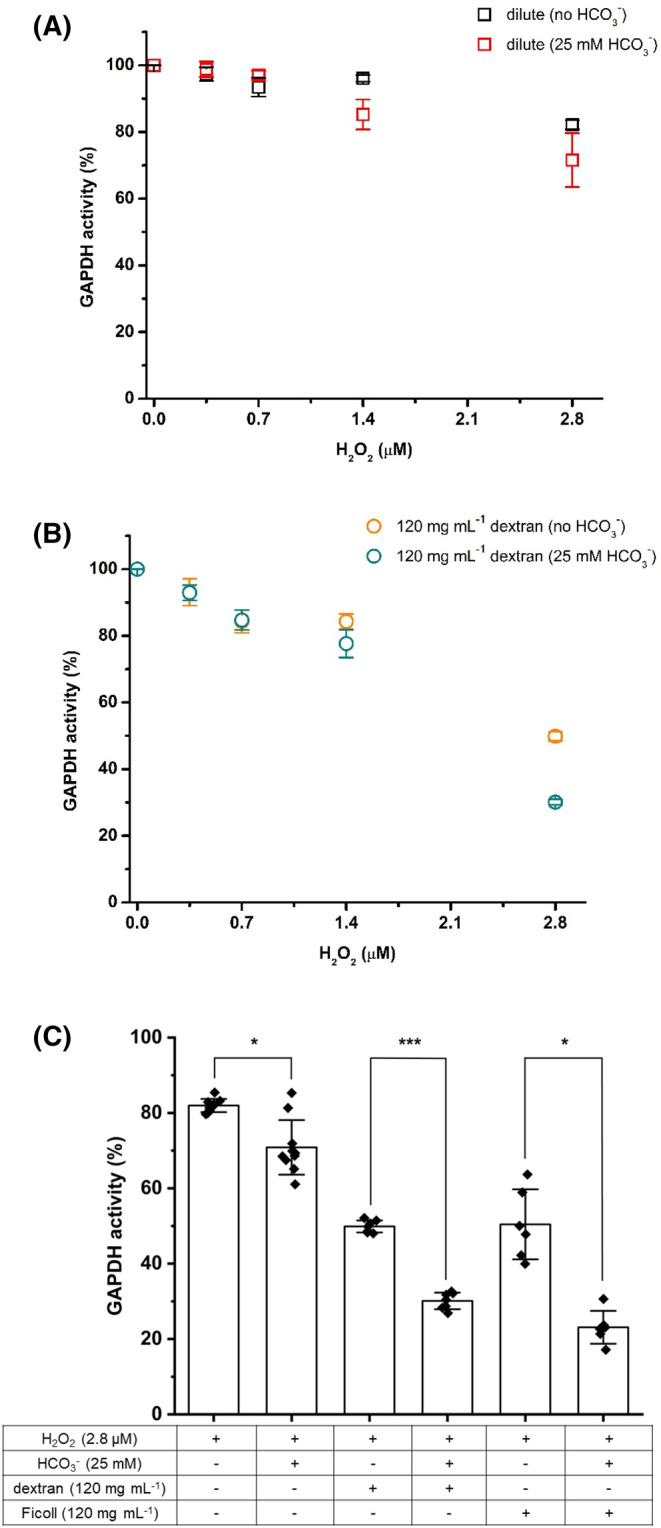
Bicarbonate (${\mathrm{HCO}}_{3}^{-}$) enhances H_2_O_2_-mediated inactivation of GAPDH under macromolecular crowding, when compared with dilute, conditions. GAPDH (0.8 μM, monomer) was incubated with 0, 0.35, 0.7, 1.4 or 2.8 μM H_2_O_2_ for 3 h at 37°C under dilute (**A**) or crowded (**B**) conditions (i.e. in the presence of 120 mg ml^−1^ dextran 35) in the absence or the presence 25 mM ${\mathrm{HCO}}_{3}^{-}$. Activity data are given as % values relative to the incubated 0 μM H_2_O_2_ incubated control. (**C**) Quantitative analysis of residual GAPDH activity after incubation with 2.8 μM H_2_O_2_ for 3 h at 37°C under dilute, 120 mg ml^−1^ dextran 35, or 120 mg ml^−1^ Ficoll 70 conditions in the absence or presence of 25 mM ${\mathrm{HCO}}_{3}^{-}$. Representative kinetic profiles of the changes in absorbance at 340 nm over time from which the GAPDH activity data was obtained for the different experimental conditions depicted in Panel C are available in the supplementary material. GAPDH activity (% values relative to the incubated 0 μM H_2_O_2_ incubated control) was determined as described in Materials and methods. Data are mean ± SD from at least six independent experiments, with statistical differences (as determined by one-way ANOVA and Dunnett's *post hoc* test) indicated as follows: ns, no statistical difference, *P < 0.05, **P < 0.01, and ***P < 0.001.

Bicarbonate has been reported previously to modulate H_2_O_2_-induced GAPDH inactivation [10]. This was confirmed under our experimental conditions. Due to the slow rate of reaction of H_2_O_2_ with CO_2_ (second-order rate constant, *k*, ∼0.02 M^−1^ s [21]) to give H_2_CO_4_/HCO_4_^−^ (pK_a_ ∼3.4 [22]), H_2_O_2_ was pre-incubated with 25 mM sodium bicarbonate in 100 mM phosphate buffer (pH 7.4) for >7 min before addition of the enzyme. An enhancement in H_2_O_2_-induced inactivation was observed in the presence of 25 mM bicarbonate (Figure 5A**)**. Thus, incubation of 0.8 µM GAPDH with 1.4 µM H_2_O_2_ in the absence or presence of 25 mM bicarbonate resulted in an activity decrease of ∼4% and ∼15%, respectively. This difference was enhanced at higher concentrations of H_2_O_2_ (2.8 µM) with a decrease in GAPDH activity of ∼18% and 28.5% detected in the absence and presence of 25 mM bicarbonate, respectively (Figure 5A).

### Examination of the combined effects of macromolecular crowding and bicarbonate on H_2_O_2_-induced GAPDH inactivation.

As both macromolecular crowding and bicarbonate appear individually to contribute to the inactivation of GAPDH induced by H_2_O_2_ (see above), further studies were carried out to determine whether these effects were alternative, additive or synergistic. Figure 5B shows the GAPDH activity (as a %) on incubation with increasing doses of H_2_O_2_ in the presence of 120 mg ml^−1^ dextran 35 in the absence, or presence, of 25 mM bicarbonate. A greater inactivation of the enzyme was detected with the presence of both bicarbonate and dextran in the reaction system. Thus, an activity of ∼50 ± 1.4% was determined for GAPDH incubated with 2.8 µM H_2_O_2_ in the presence of 120 mg ml^−1^ dextran 35, and an activity of ∼31 ± 0.8% was determined for system containing both 120 mg ml^−1^ and 25 mM bicarbonate during GAPDH exposure to H_2_O_2_ (Figure 5C). Incubation of GAPDH with 2.8 µM H_2_O_2_ in the presence of Ficoll 70 had a similar effect, with an enzyme activity of ∼50 ± 9.1% with only the crowding agent present (Figure 3) and a residual activity of ∼23 ± 4.2% with both Ficoll 70 and bicarbonate present (Figure 5C).

## Discussion

GAPDH plays an important role in the metabolic adaptation of cells undergoing oxidative stress [3], as GAPDH inactivation re-routes the carbon flux from glycolysis to the oxidative branch of the pentose phosphate pathway, thereby enhancing the production of reducing equivalents (in the form of NAPDH) and synthetic intermediates that provide tolerance of damage by way of an enhanced capacity of antioxidant defense and repair systems [23]. However, an open question is to how GAPDH is efficiently inactivated in the crowded cytosol of cells where the total amount of proteins ranges between 2 and 4 million proteins per µm^3^ [24], and the average concentration of intracellular proteins thiols is 10–40 mM [25]. In particular, this enzyme would not be expected, based on prior kinetic data (see Introduction), to be readily inactivated due to the slow rate of reaction of H_2_O_2_ with GAPDH. Different hypotheses have been proposed to rationalize this conundrum, and explain how reaction with H_2_O_2_ (or other oxidants) might enable its activity as a redox switch. Redox relays have been proposed as a possible explanation (i.e. inactivation of GAPDH via the intervention of another protein with which H_2_O_2_ reacts much more rapidly). Such processes have been identified for a number of other proteins that act as ‘redox switches’ (e.g. oxidation of STAT3 via oxidized peroxiredoxin-2, thioredoxin/ASK1 by oxidized peroxiredoxin-1 and others; [26,27]), but GAPDH has not been shown (so far) to be selectively oxidized via this type of process. However, a recent study has reported that inactivation of GAPDH can be mediated by superoxide dismutase (SOD1) [28]. This is believed to occur via a proximity-model (i.e. due to interactions between the enzymes) in which the generation of localized H_2_O_2_ by SOD1 may induce efficient oxidation of GAPDH. In addition, it has been proposed that the composition of the reaction media may be of relevance as bicarbonate, an important buffering agent in the cell cytosol, can modulate the reactivity of H_2_O_2_, via the formation of the more reactive adduct species peroxymonocarbonate, ${\mathrm{HCO}}_{4}^{-}$ [21]. Consistent with this proposal, inactivation of GAPDH by H_2_O_2_ has been shown to be enhanced in the presence of bicarbonate, via a mechanism where GAPDH facilitates *in situ* formation of ${\mathrm{HCO}}_{4}^{-}$ [10].

We have recently reported that macromolecular crowding, an intrinsic property of biological systems, can modulate the mechanism(s) of reaction of H_2_O_2_ with GAPDH, with the data obtained consistent with a greater extent of hyperoxidation of the active site Cys residue under crowded, compared with dilute conditions [17]. In the light of these data, we investigated the effects of macromolecular crowding, bicarbonate, and a mixture of these (which is likely to most accurately reflect the *in vivo* situation) on the activity of GAPDH.

Low concentrations of H_2_O_2_ (0.8 µM GAPDH with 0.7 µM H_2_O_2_) under dilute conditions had no significant effects on GAPDH activity, in agreement with previous reports [10,29] (Figure 1). Higher concentrations of H_2_O_2_ (2.8 µM H_2_O_2_ corresponding to a 3.5-fold molar excess over the protein concentration), gave modest, but significant, extents of inactivation, consistent with the established kinetic data. This range of H_2_O_2_ concentrations was then used to determine whether macromolecular crowding and/or bicarbonate enhance inactivation. Dextran 35 (final concentration 60 mg ml^−1^ when the activity of the enzyme was examined) did not alter the kinetics of reduction of NAD^+^ by GAPDH (as measured by the absorbance increase at 340 nm; Figure 2A) with this data being in agreement with previous data suggesting that macromolecular crowding does not alter the specific activity of GAPDH [20]. This lack of effect of dextran 35 at the moment of determining the enzyme activity was also observed for samples of GAPDH previously oxidized under dilute conditions (Figure 2B). Thus, no differences in residual activity (% values compared with controls) were observed for samples oxidized under dilute conditions, when the activity measurements were determined in the absence or presence of the crowding agent. However, when the exposure to H_2_O_2_ was carried out under crowded conditions (i.e. in the presence of 120 mg ml^−1^ dextran 35) an enhanced loss of the activity was detected (Figure 2B,C). Similar behavior was observed across all the H_2_O_2_ concentrations tested (0.35–2.8 µM). These novel data indicate that crowding modulates both the oxidation mechanism (as reported in [17]), and also enzyme activity. This effect of macromolecular crowding can be rationalized by increased interaction between small molecules (in this case H_2_O_2_) and the target protein [30]. It has been reported that besides an increased susceptibility of the active site Cys (Cys150 in the rabbit isoform) to oxidation, the catalytic site of GAPDH possesses a binding site for H_2_O_2_ in the shallow cavity formed by this Cys and neighboring proton-donating residues [7]. Thus, macromolecular crowding may contribute to interactions that decrease the activation energy by stabilizing the transition state formed during the (two-electron nucleophilic) attack of the thiol of the catalytic Cys on H_2_O_2_, thereby increasing the rate of this reaction.

Similar experiments with the branched polymer Ficoll 70 (Mw ∼70 000 g mol^−1^), which has similar chemical properties to (linear) dextran 35, with both being polyols, demonstrated that this enhanced inactivation is not particular to dextran (Figure 3). This general phenomenon appears to arise from the excluded volume phenomenon as high concentrations of sucrose (Mw 342 g mol^−1^), which enhances solution viscosity but would not otherwise be expected to impede interactions in the same manner as a large polymer, had minimal effects on H_2_O_2_-induced GAPDH inactivation (Figure 4). However, it should also be considered that the large steric bulk of dextran 35 or Ficoll 70 may alter GAPDH structure dynamics, and may enhance weak attractive forces. Similar findings have been reported for complexes formed between bovine serum albumin and poly(diallyldimethylammonium chloride) where the crowding agent polyethylene glycol (Mw ∼8000 g mol^−1^) enhanced complex formation, whilst sucrose did not [31].

The current experiments have confirmed that the presence of 25 mM bicarbonate, under the conditions employed, enhances H_2_O_2_-induced GAPDH inactivation under dilute conditions (absence of crowding agent; Figure 5A). The additional presence of dextran 35, to induce macromolecular crowding, resulted in a greater loss of enzyme activity when compared with the absence of bicarbonate (Figure 5B,C). Thus, after incubation of GAPDH (0.8 µM) with 2.8 µM H_2_O_2_ residual activity levels of ∼50% and ∼30% were determined under macromolecular crowding conditions in the absence and the presence of 25 mM bicarbonate, respectively (Figure 5C). Similar data was obtained when Ficoll was used as the crowding agent.

Despite these novel observations, a number of important, but as yet unresolved, questions remain to be explored including: (1) whether (and how) macromolecular crowding modulates the *in situ* formation of ${\mathrm{HCO}}_{4}^{-}$ by GAPDH, as suggested by Winterbourn et al. [10]; (2) whether macromolecular crowding modulates the effects of both the GAPDH substrate (G3P) and cofactor (NAD^+^) on H_2_O_2_-induced inactivation, and particularly in the presence of bicarbonate; and (3) whether macromolecular crowding and bicarbonate alter the oxidation pathways of GAPDH [17].

## Conclusions

The data presented here demonstrate that both macromolecular crowding and buffer composition enhance H_2_O_2_-induced GAPDH inactivation, with the combination of these factors resulting in additive effects. These data may help explain how GAPDH is efficiently modified and inactivated in the cytosol of cells under conditions of oxidative stress, with these changes being consistent with its role as a metabolic switch under (patho)physiological conditions [3]. These data suggest that the second-order rate constant reported for reaction of H_2_O_2_ with GAPDH, determined under dilute conditions, may underestimate the rate constant for enzyme oxidation under biological conditions, by a considerable margin. Further experiments appear to be warranted to determine the magnitude of this difference in rate constants, though such measurements are likely to be complicated by the non-homogeneous effects that occur under crowded conditions, which may hinder traditional kinetic analyses.

## Materials and methods

### Reagents

GAPDH (rabbit muscle), catalase (bovine liver), G3P, NAD^+^, H_2_O_2_, dextran 35 (average molecular mass 35 000–45 000 g mol^−1^), sucrose, glycerol, diethylenetriaminepentaacetic acid (DTPA) and other biochemicals were from Sigma–Aldrich (Søborg, Denmark). Ficoll® PM70 (average molecular mass ∼70 000 g mol^−1^) was purchased from VWR (Søborg, Denmark). H_2_O_2_ was standardized by measuring its absorbance at 240 nm using a molar absorptivity of 39.4 M^−1^ cm^−1^. To generate ${\mathrm{HCO}}_{4}^{-}$, H_2_O_2_ was diluted into bicarbonate-containing buffer (25 mM sodium bicarbonate in 100 mM sodium phosphate buffer, pH 7.4, containing 0.1 mM DTPA), and incubated for at least 7 min before use. All solutions were prepared using ultrapure water.

### GAPDH oxidation by H_2_O_2_

GAPDH (0.8 µM in monomer units) was incubated with 0.35, 0.7, 1.4 or 2.8 µM H_2_O_2_ (i.e. 0.4–3.5-fold molar excess over the GAPDH monomer concentration) for 3 h at 37°C in 100 mM sodium phosphate buffer (pH 7.4) containing 0.1 mM DTPA. For the experiments carried out in the presence of sodium bicarbonate (25 mM), buffers were prepared on the day of the experiment with the pH adjusted to 7.4 before use. The solutions used in the experiments under crowded conditions were prepared by mixing stock solutions prepared in 100 mM sodium phosphate buffer (pH 7.4) containing 0.1 mM DTPA and 120 mg ml^−1^ dextran 35, Ficoll® 70, sucrose or glycerol. The reaction volume was typically between 50 and 100 µl, with the oxidation reactions stopped at the required time points by adding catalase (20 µg ml^−1^) to remove excess H_2_O_2_.

### Assessment of GADPH activity

GAPDH activity was measured by diluting control and treated samples to an enzyme concentration of 0.4 µM (i.e. 1:1 dilution) in 100 mM Tris buffer (pH 8.0) containing 0.4 mM G3P, 1 mM NAD^+^, 15 mM sodium arsenate and 0.2 mM EDTA in Greiner UV-Star® 96 well plates (VWR) [10]. Thus, for samples prepared under crowded conditions (i.e. in the presence of 120 mg ml^−1^ dextran 35, Ficoll® 70, sucrose, or glycerol), a final concentration of 60 mg ml^−1^ of the crowding agents was present when the activity was measured. Initial slopes were determined from the increase in absorbance at 340 nm due to NADH formation using a SpectraMax® i3 plate reader (Molecular Devices, U.S.A.). Activity data are presented as a percentage relative to the respective control (i.e. incubation in the absence of H_2_O_2,_ and/or bicarbonate, under both dilute and crowded conditions). Plate readings were performed separately for each experimental condition to avoid any lag time between the addition of substrate to the enzyme and the start of the plate reading.

### Statistical analysis

Activity data are presented as mean ± SD from at least six individual experiments, with the figures created using OriginPro. Statistical significance was determined by one-way ANOVA using Dunnett's *post hoc* test. Significance was assumed at the *P* < 0.05 level.

## Data Availability

Experimental data will be made available on reasonable request to the authors.

## Abbreviations

GAPDH: glyceraldehyde-3-phosphate dehydrogenase
SOD1: superoxide dismutase
