# Supplementary material for: Macromolecular crowding and bicarbonate enhance the hydrogen peroxide-induced inactivation of glyceraldehyde-3-phosphate dehydrogenase
Source: Biochem J. 2024 Dec 4;481(23):1855–66. doi: 10.1042/BCJ20240597 (PMC11668361; doi:10.1042/BCJ20240597)
Supplement: Supplementary Material [file BCJ-481-1855-s1.pdf]

Supplementary material for

Macromolecular crowding and bicarbonate enhance the hydrogen peroxide-induced inactivation of glyceraldehyde-3-phosphate dehydrogenase

Rebecca H. J. Bloemen [1], Rafael Radi [2,3], Michael J. Davies [1 §] and Eduardo Fuentes-Lemus [1,4 § *]

[1] Department of Biomedical Sciences, Panum Institute, University of Copenhagen, Blegdamsvej 3, Copenhagen, 2200, Denmark.

[2] Departamento de Bioquímica, Facultad de Medicina, Universidad de la República, Montevideo, 11800, Uruguay.

[3] Centro de Investigaciones Biomédicas (CEINBIO), Facultad de Medicina, Universidad de la República, Montevideo, 11800, Uruguay.

[4] Departamento de Química Física, Facultad de Química y de Farmacia, Pontificia Universidad Católica de Chile, Santiago, Chile.

§ Joint senior authors.

* To whom correspondence should be addressed: eduardo.lemus@sund.ku.dk or effuente@uc.cl.

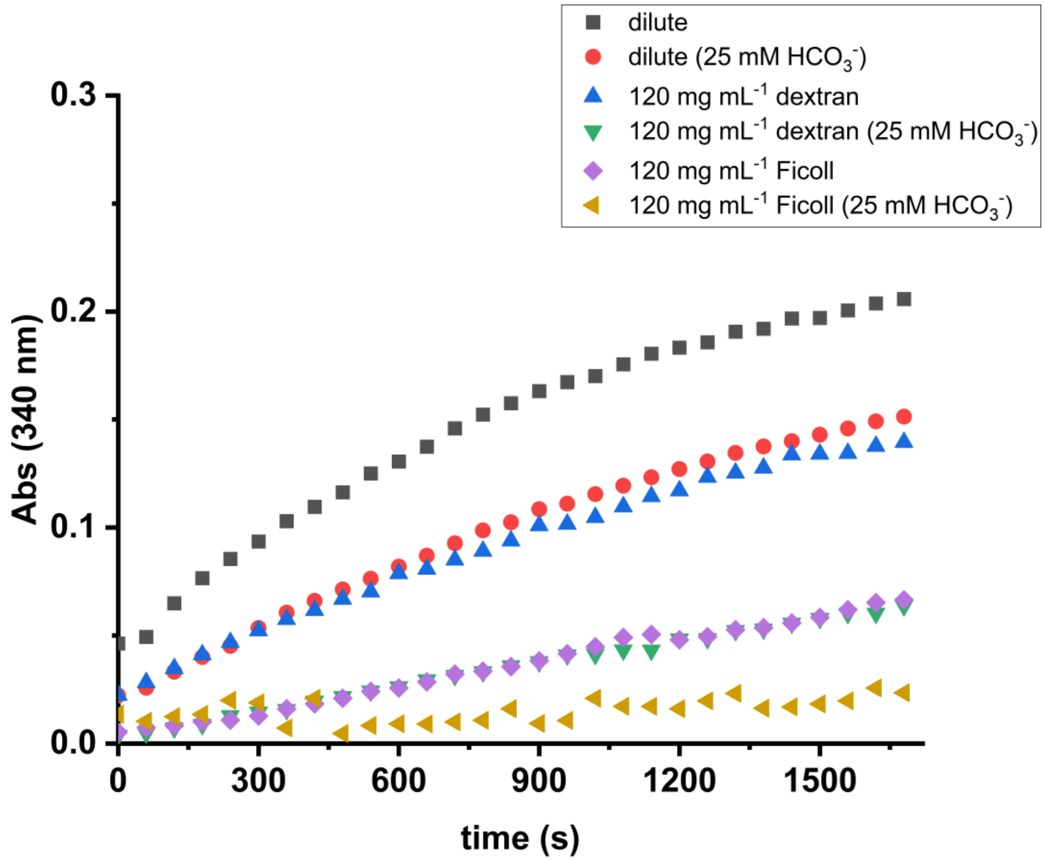

Supplementary Figure 1: Changes in absorbance at 340 nm, arising from NADH formation during the incubation of 0.2 mM G3P and 0.5 mM NAD^+ with GAPDH samples pre-incubated with 2.8 μM H_2O_2 in the absence or the presence of 25 mM bicarbonate (HCO_3^-) under dilute or crowded (120 mg mL^{-1} dextran or Ficoll) conditions. Representative data from independent experiments from which GAPDH activities were determined for **Fig. 5**.
